# Towards the third dimension in direct electron beam writing of silver

**DOI:** 10.3762/bjnano.9.78

**Published:** 2018-03-08

**Authors:** Katja Höflich, Jakub Mateusz Jurczyk, Katarzyna Madajska, Maximilian Götz, Luisa Berger, Carlos Guerra-Nuñez, Caspar Haverkamp, Iwona Szymanska, Ivo Utke

**Affiliations:** 1 Empa - Swiss Federal Laboratories for Materials Science and Technology, Laboratory for Mechanics of Materials and Nanostructures, Feuerwerkerstrasse 39, 3602 Thun, Switzerland; 2 Helmholtz-Zentrum Berlin für Materialien und Energie, Nanoscale Structures and Microscopic Analysis, Hahn-Meitner-Platz 1, 14109 Berlin, Germany; 3 Faculty of Physics and Applied Computer Sciences, AGH University of Science and Technology, 30-059 Kraków, Poland; 4 Faculty of Chemistry, Nicolaus Copernicus University in Toruń, Gagarina 7, 87-100 Toruń, Poland

**Keywords:** carboxylate, electron beam induced deposition, silver, three-dimensional nanostructures, vertical growth rate

## Abstract

Carboxylates constitute an extremely promising class of precursor compounds for the electron beam induced deposition of silver. In this work both silver 2,2-dimethylbutyrate and silver pentafluoropropionate were investigated with respect to their dwell-time-dependent deposition behavior and growth characteristics. While silver 2,2-dimethylbutyrate showed a strong depletion in the center of the impinging electron beam profile hindering any vertical growth, silver pentafluoropropionate indicated a pronounced dependency of the deposit height on the dwell time. Truly three-dimensional silver structures could be realized with silver pentafluoropropionate. The pillars were polycrystalline with silver contents of more than 50 atom % and exhibit strong Raman enhancement. This constitutes a promising route towards the direct electron beam writing of three-dimensional plasmonic device parts from the gas phase.

## Introduction

Focused electron beam induced deposition (FEBID) is a resistless direct-write technique that allows for a highly precise fabrication of three-dimensional nanostructures [1–2]. Gaseous precursor molecules are injected into the vacuum chamber of a scanning electron microscope and are locally dissociated by a focused electron beam [3]. After dissociation, the non-volatile part forms the deposit while the volatile rest is pumped out. The dissociation is a complex process influenced by the local dynamics of the precursor molecules and induced by electrons with their specific, yet mostly unknown, cross-sections for the respective energy ranges and molecule bonds to break [3–4]. The used primary electrons of energies in the kiloelectronvolt range have a focal spot of several nanometers or even less. Secondary electrons are generated by both primary and back-scattered electrons and hence escape in a radius of up to several micrometers [5]. Mainly these low-energy electrons are expected to contribute to the precursor dissociation [4]. The ultimate resolution of the fabricated features strongly depends on the number and energy of primary electrons [6–7]. In this respect, the vertical growth rate plays a crucial role. The vertical growth rate is determined by the precursor dynamics, especially by adsorption and by diffusion of the molecules, and by the actual precursor flux. Upon vertical growth, the size of the interaction volume where secondary electrons are generated, significantly decreases since it moves upwards into the deposit [8]. If the vertical growth rate is small, secondary electrons emerging from the surrounding substrate and the lower parts of the deposit strongly contribute to the precursor dissociation. In an extreme case, this even hinders the evolution of nanostructures. Instead, a pronounced halo of a diameter in the micrometer range occurs [9].

For the deposition of metals, typically metal-organic precursor compounds are employed [3]. The organic ligands bring the desired metal into the gas phase. Hence, a sufficiently high stability and vapor pressure is usually accompanied by a large amount of carbon in the compound [10]. This carbon is incorporated into the deposit by ligand co-dissociation together with the dissociated residual hydrocarbons from the vacuum background [11]. Pure material could be deposited only in few cases in which an inorganic precursor compound [12–13] or catalytic activity [14] was utilized.

The chemical reactions occurring during deposition can be elucidated by surface-science studies in which low-energy electrons dissociate monolayers of precursors under ultra-high vacuum conditions [4]. Based on these results the design of precursors for new materials and enhanced purity of the deposits is conceivable [15–16]. The identification of such novel precursor compounds for FEBID is a subject of intense research since direct writing of 3D materials and nanodevices can advance diverse applications, for example in the field of plasmonics [17–19]. One ideal plasmonic material is silver, which exhibits strongly resonant behavior in the visible range without suffering from losses due to interband transitions [20]. However, the coinage metal silver comes with some technical issues. It tends to react to silver sulfide under ambient conditions [21] and hence requires an encapsulation within the final device. Furthermore, the electron beam induced deposition of silver is challenging. Many potential precursor candidates have to be heated above 100 °C and show extremely low vapor pressures [22–25]. This is related to the main oxidation state of +1 for silver, which severely limits the possibility to attach appropriate ligands. Even more importantly, the ligands tend to be only weakly bonded and, thus, easily exchange the metal atom [26]. These properties exclude the gas-phase FEBID of silver for conventional gas-injection systems (GIS) that are flanged at the outer chamber walls.

Recently, the first gas-phase silver FEBID could be realized with a fully integrated GIS [9]. The used compound silver 2,2-dimethylbutyrate [Ag(µ-O_2_CC(CH_3_)_2_CH_2_CH_3_)]_2_ (AgO_2_Me_2_Bu) is extremely sensitive to electron beam impact. Deposition suffers from its low vapor pressure and its low vertical growth rate. Consequently, no growth of silver nanostructures could be achieved. To address these issues AgO_2_Me_2_Bu was compared to another carboxylate compound. Silver pentafluoropropionate [Ag(µ-O_2_CC_2_F_5_)]_2_ (AgO_2_F_5_Prop) provides for a similar evaporation temperature and electron-beam sensitivity but also for slightly higher gas flux and stability leading to deposits of high silver contents [27]. This makes it ideally suited for a comparative study elucidating the deposition behavior of such carboxylate compounds. Depositions varying beam and current and dwell times were investigated concerning their morphology and composition. Finally, FEBID of truly three-dimensional silver structures could be realized for the first time. The resulting pillars exhibit large silver contents of more than 50 atom %.

## Experimental

Depositions were carried out onto n-doped silicon wafers in a Hitachi S 3600 tungsten-filament microscope equipped with a fully integrated custom-built gas-injection system (GIS). The GIS was designed for short molecule paths and chemical inertness to allow for the evaporation of highly reactive compounds with low vapor pressure. The GIS three-axis stage allowed for accurate positioning of the nozzle exit 200 µm above the sample surface. The stage was heated by a resistive heating element up to a temperature of 130–160 °C measured inside the copper block carrying the sample. The samples were clamped onto the copper block. The resulting temperature gradient leads to a temperature of 100–130°C onto the sample surface.

Silver 2,2-dimethylbutyrate [Ag(µ-O_2_CC(CH_3_)_2_CH_2_CH_3_)]_2_, CAS 1085717-13-0 (AgO_2_Me_2_Bu) and silver pentafluoropropionate [Ag(µ-O_2_CC_2_F_5_)]_2_, CAS 509-09-1 (AgO_2_F_5_Prop) were used as precursors for silver deposition. The compounds were synthesized according to previously reported procedures [24,28]. In the case of silver 2,2-dimethylbutyrate, carboxylic acid and potassium nitrate were suspended in a water–ethanol solution, heated up to 40 °C and stirred, followed by the addition of silver nitrate. Silver pentafluoropropionate was synthesized by the reaction of fluorinated carboxylic acid and silver carbonate.

The experiments started with reproducing the results from AgO_2_Me_2_Bu using a GIS heating temperature of 150 °C. In case of AgO_2_F_5_Prop, earlier studies [27] showed the successful deposition for a GIS temperature of 175 °C and a substrate temperature of 160 °C. To minimize unwanted thermal effects during and after deposition these temperatures were decreased to 140 °C GIS temperature and ca. 155 °C stage temperature (the latter being equivalent to ca. 125 °C substrate temperature). The used GIS temperature still ensured the full evaporation of AgO_2_F_5_Prop with a mass loss of 3 mg per hour. This finally resulted in a gas flux of around 30 × 10^15^ molecules per second and cm^2^ roughly doubling the typical gas flux of AgO_2_Me_2_Bu [9]. Due to the low vapor pressure of the compounds, the growth pressure equaled the base pressure being typically around 3 × 10^−5^ hPa.

A Xenos Patterning engine was used to define the patterning parameters. Square patterns of 10 × 10 µm^2^ with a pitch of 3 nm and 100 repeats were written using a spiral beam path and 500 pA beam current. Spot arrays with 5 µm distance between the respective spots were exposed with increasing dwell times from row to row and 100,000 repeats. For the dwell time study and the pillar growth beam currents of 50, 150 and 500 pA were used. These correspond to FWHM of the primary electron beam of 100, 200 and 350 nm, respectively, as determined by imaging of lacey carbon edges. The acceleration voltage was kept constant at 15 kV throughout the whole deposition series.

High-resolution scanning electron microscopy (SEM) imaging and energy-dispersive X-ray spectroscopy (EDX) was performed using a Hitachi S 4800 equipped with an EDAX silicon drift detector (SSD). EDX data acquisition was performed for acceleration voltages of 8 and 12 kV using a sample current of around 5 nA with a take-off angle of 42° and 30 s acquisition time. The two voltages yielded two independent sets of data for the extraction of the *k*-ratios of each atom. After background subtraction these *k*-ratio values together with the deposit thicknesses served as input for the SAMx STRATAGem thin film analysis. This allowed for the quantification of the atomic composition inside the deposit excluding the signal contribution from the substrate. Since carbon deposition due to residual background gases always occurs during EDX signal acquisition, this carbon content was determined using a reference silver layer. The quantified carbon background of ca. 15 atom % was subtracted to determine the actual deposit composition. The topography of the deposits was monitored using atomic force microscopy (AFM) with an AIST Smart SPM system. Data processing was carried out using the free software Gwyddion 2.49. Confocal Raman spectroscopy was performed using an upright ND-MDT NTEGRA Raman microscope featuring a laser source with a wavelength of 532 nm and a 100× objective lens with a numerical aperture of 0.95. Spectra were recorded at a spectral resolution of 2.7 cm^−1^ with 5 s exposure time for each deposit. Graphical data were processed using OriginPro 2017G.

## Results and Discussion

**Figure 1:**
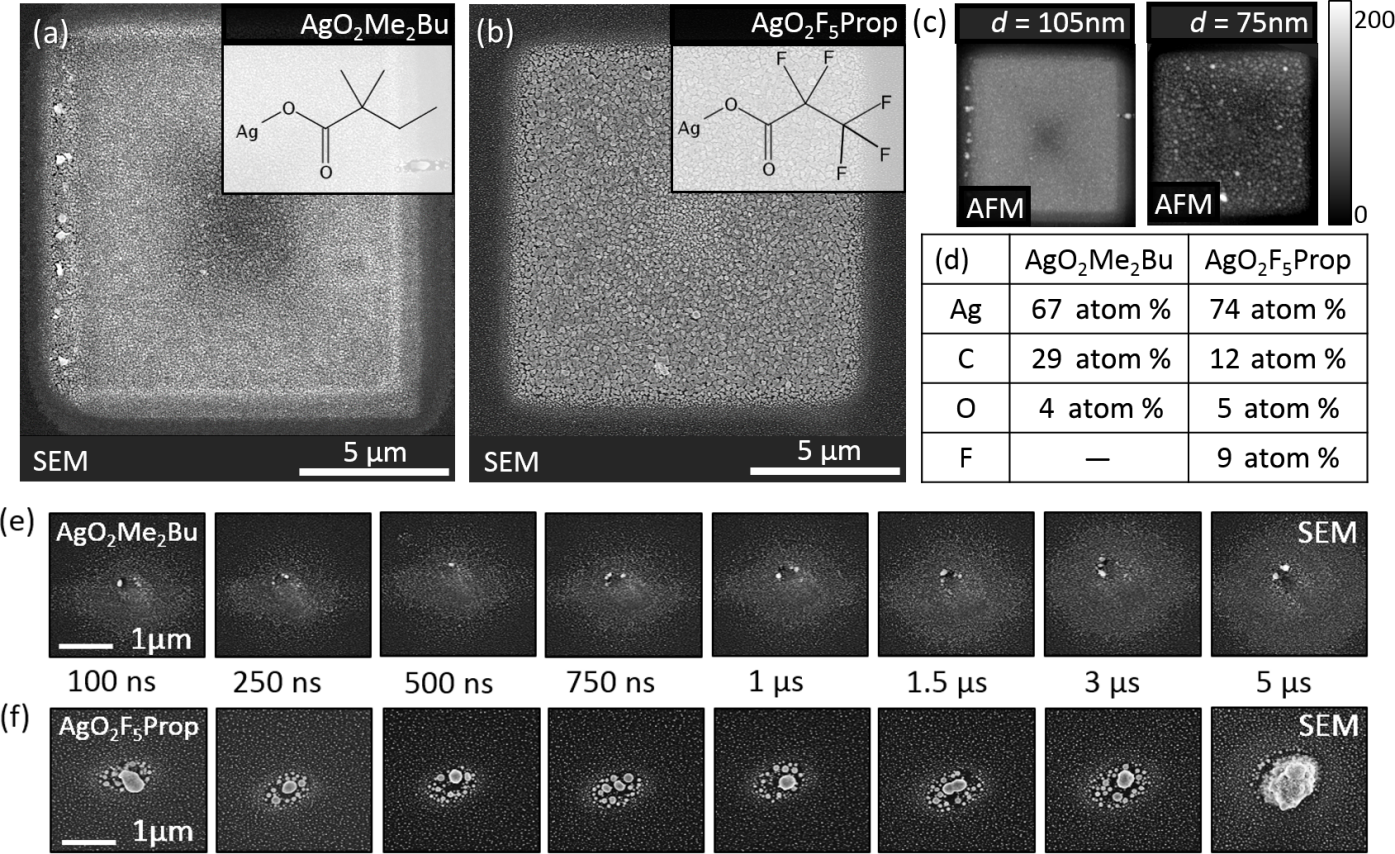
Scanning electron micrographs of deposits from (a, e) AgO_2_Me_2_Bu and (b, f) AgO_2_F_5_Prop using a beam current of 500 pA. (c) The deposit heights of the square deposits in (a) and (b) served as input for the thin-film correction of the EDX data leading to the elemental composition shown in the table in panel (d). Spot deposits with varying dwell times are displayed in (e) and (f). AgO_2_Me_2_Bu deposits show depletion in the beam center becoming more and more pronounced for increasing dwell times. In contrast, AgO_2_F_5_Prop shows an enhanced growth in the central region.

Figure 1 shows the deposition results for a beam current of 500 pA. The square patterns of 10 µm side length in Figure 1a,b were fabricated using 1 µs dwell time and a pitch of 3 nm. The insets depict the molecular structure of the precursors used. AgO_2_F_5_Prop contains only half of the amount of carbon compared to AgO_2_Me_2_Bu but a large portion of fluorine instead. Besides that the molecular structure is very similar, which leads to the expectation that also AgO_2_F_5_Prop will show a strong sensitivity to electron beam impact. Indeed, this could be proven by earlier investigations [27].

For the shown case of slow electron-beam movement, AgO_2_F_5_Prop and AgO_2_Me_2_Bu showed comparable growth rates. As visible from Figure 1c, the deposit height for AgO_2_Me_2_Bu was larger with 105 nm compared to 75 nm although the precursor flux is slightly lower. The square deposits show the earlier observed surface roughness for both of the precursors. The corresponding EDX quantification points to an even higher silver content in case of the AgO_2_F_5_Prop precursor, while the small amount of oxygen is comparable. The determined silver content of 74 atom % for AgO_2_F_5_Prop is remarkable but accompanied by a detectable amount of the fluorine incorporated into the deposit. The amount of fluorine in the deposit roughly equals the amount of incorporated carbon.

To elucidate the precursor dynamics, spot exposures using a beam current of 500 pA with varying dwell times were carried out. The dwell time was increased from 100 ns as the minimum value that could be realized with the patterning engine up to 5 µs to cover the typical time scale for precursor depletion. At the end of each line, the beam dwells for approximately 5 ms to account for a full refreshment of the surface with precursor molecules. (This time value was back-calculated from the total deposition times and corresponds to internal software time delays that are not directly accessible. At this end position the beam was not blanked.) The SEM images in Figure 1e,f show the significant differences in the deposition behavior for spot deposits of both precursors. According to the deposition regimes proposed earlier [9] the deposits for AgO_2_Me_2_Bu in Figure 1e show a suppressed deposition in the central beam region of high electron flux. This is attributed to poisoning of the silver crystals by ligand co-dissociation under low precursor flux [9]. Around the beam tails, where the number of generated secondary electrons is decreased by orders of magnitude, the formation of silver crystals is observed. In contrast, the silver crystal formation is especially pronounced in the central region of the beam focus for the case of AgO_2_F_5_Prop. This is most probably not a consequence of higher flux of precursor gas since the density of molecules escaping from the gas nozzle has the same order of magnitude for both compounds.

Given the same beam conditions in both experiments the number of molecules that is finally dissociated in the focal region could otherwise only differ due to a different adsorption behavior of both species. Besides, a strongly weakened poisoning effect for the case of AgO_2_F_5_Prop cannot be excluded at this point. With a ratio of Ag/C = 1:3, AgO_2_F_5_Prop contains only half of the carbon content in its stoichiometry compared to AgO_2_Me_2_Bu. Since the poisoning is expected to be caused mainly by the co-dissociation of carbon, this may explain the enhanced growth in the beam center for the case of AgO_2_F_5_Prop.

**Figure 2:**
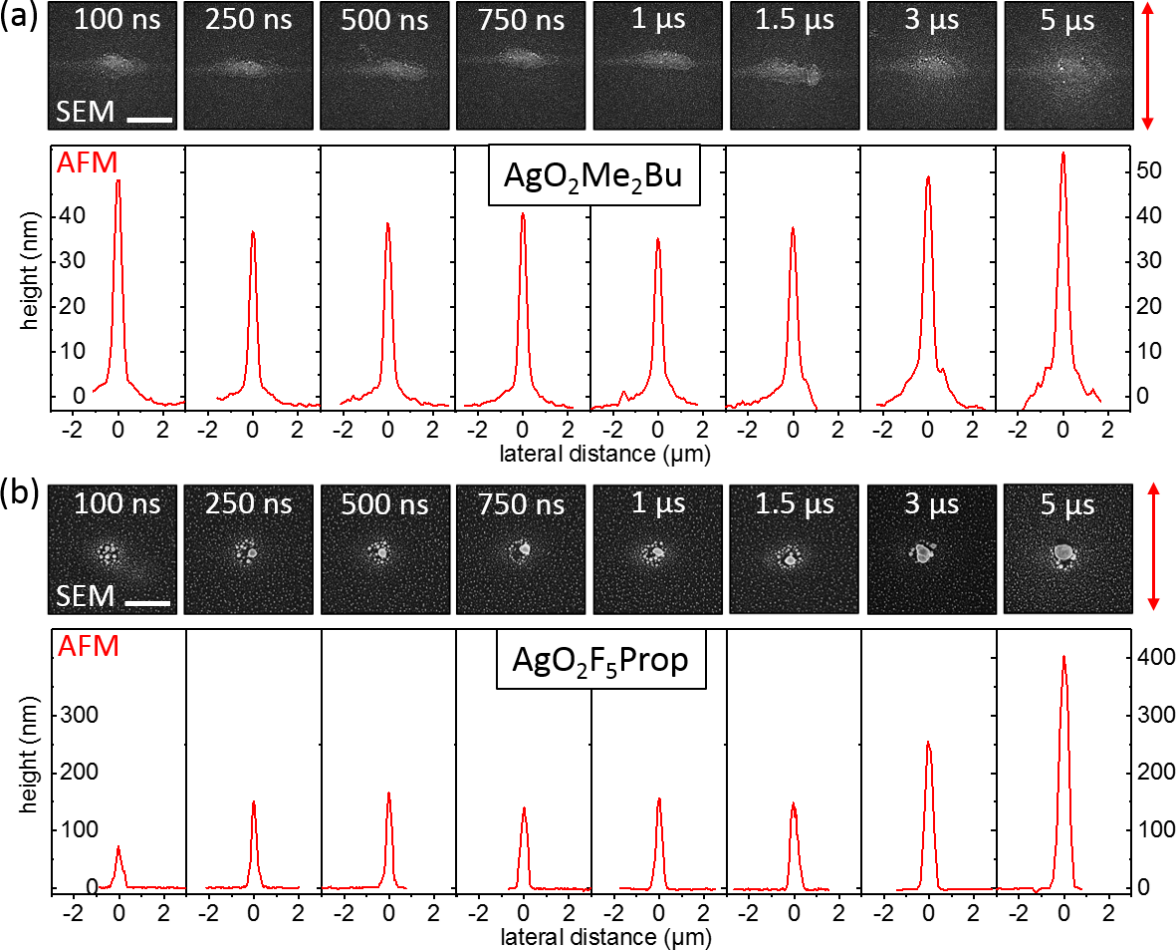
Dwell-time series for a beam current of 150 pA using (a) AgO_2_Me_2_Bu and (b) AgO_2_F_5_Prop as precursor compounds. The upper rows of panels (a) and (b) show scanning electron micrographs of the spot deposits with the red arrow indicating the direction of taking AFM profiles. Below the SEM images averaged height profiles are displayed. While there is no distinct correlation between dwell time and deposit height for AgO_2_Me_2_Bu, the deposit height significantly increases for AgO_2_F_5_Prop. The red arrow on the right side depicts the direction in which the AFM profiles were taken. This was chosen such, that deposit deformations due to stage drift and beam blanker velocity do not influence the displayed topography below. The AFM profiles are the result of averaging over seven deposits with the same dwell time, produced in the same experiment.

In view of these results, there is one interesting question: Does AgO_2_Me_2_Bu also show an increased growth in the electron beam focus, when the number of incident electrons is accordingly reduced to finally match the number of impinging molecules. Hence, further experiments with beam currents of 150 and 50 pA were carried out. The electron density for 500 pA is around 6 × 10^18^ s^−1^·cm^−2^ compared to 5 × 10^18^ s^−1^·cm^−2^ for 150 pA and 4 × 10^18^ s^−1^·cm^−2^ for 50 pA beam current. Still, in all three cases the electron flux exceeds the local flux of precursor molecules by more than two orders of magnitude. Hence, continuous exposure would result in an adsorbate-limited deposition regime. Dependent on the actual dwell time of the beam, co-dissociation of non-desorbed ligands as well as of residual carbon hydrates from the vacuum background is expected. Figure 2 displays results for a dwell time series using a 150 pA beam for both precursor compounds. The upper rows in Figure 2a,b show SEM images of the deposits from AgO_2_Me_2_Bu and AgO_2_F_5_Prop for dwell times increasing from 100 ns up to 5 µs. The chosen dwell time range covers the typical time scale for electron-limited and adsorbate-limited depletion regimes.

For the case of AgO_2_Me_2_Bu, the SEM images in Figure 2a show slight horizontal deformation, most probably caused by the nonzero closing time of the beam blanker. The AFM profiles displayed below show no distinct correlation between deposit height and dwell time. The mean deposit height is approximately 50 nm. For higher dwell times the halo formation becomes more pronounced and slight crystal formation occurs. In contrast to the deposits for 500 pA, no black central region occurred, which may provide a hint to precursor supply by diffusion. Still, the vertical growth rate for AgO_2_Me_2_Bu is low. Interestingly, even taking into account the halo, the deposit volume only shows a slight increase for the higher dwell times. An increase of the dwell time by a factor of 5 from 1 to 5 µs only leads to an increase in volume by a factor of around 2.5. If the low deposition rate is caused by enhanced desorption of the molecules, lowering the stage temperature could provide for enhanced vertical growth. However, already a stage temperature of 10–15 K less triggers the condensation of AgO_2_Me_2_Bu onto the substrate.

A completely different behavior was observed for AgO_2_F_5_Prop as shown in Figure 2b. Both, height and volume of the deposits increase strongly with increasing dwell time. The mean deposit heights increased from smaller than 100 nm for 100 ns dwell time up to 400 nm for 5 µs dwell time. The deposit volumes were estimated by calculating the solid of revolution for the averaged profiles around the height axis. They are used as a measure for the efficiency of the deposition. For a dwell time of 1 µs the deposit volume amounts to 1.8 × 10^−2^ µm^3^. This almost matches the corresponding volume for AgO_2_Me_2_Bu with 1.9 × 10^−2^ µm^3^. However, increasing the dwell time again by a factor of five leads to a five times higher deposit volume of around 0.1 µm^3^. While a full interpretation of these results, e.g., in terms of enhanced diffusion and less surface poisoning requires further experimental evidence, they are extremely promising in view of a fabrication of silver nanostructures using AgO_2_F_5_Prop.

**Figure 3:**
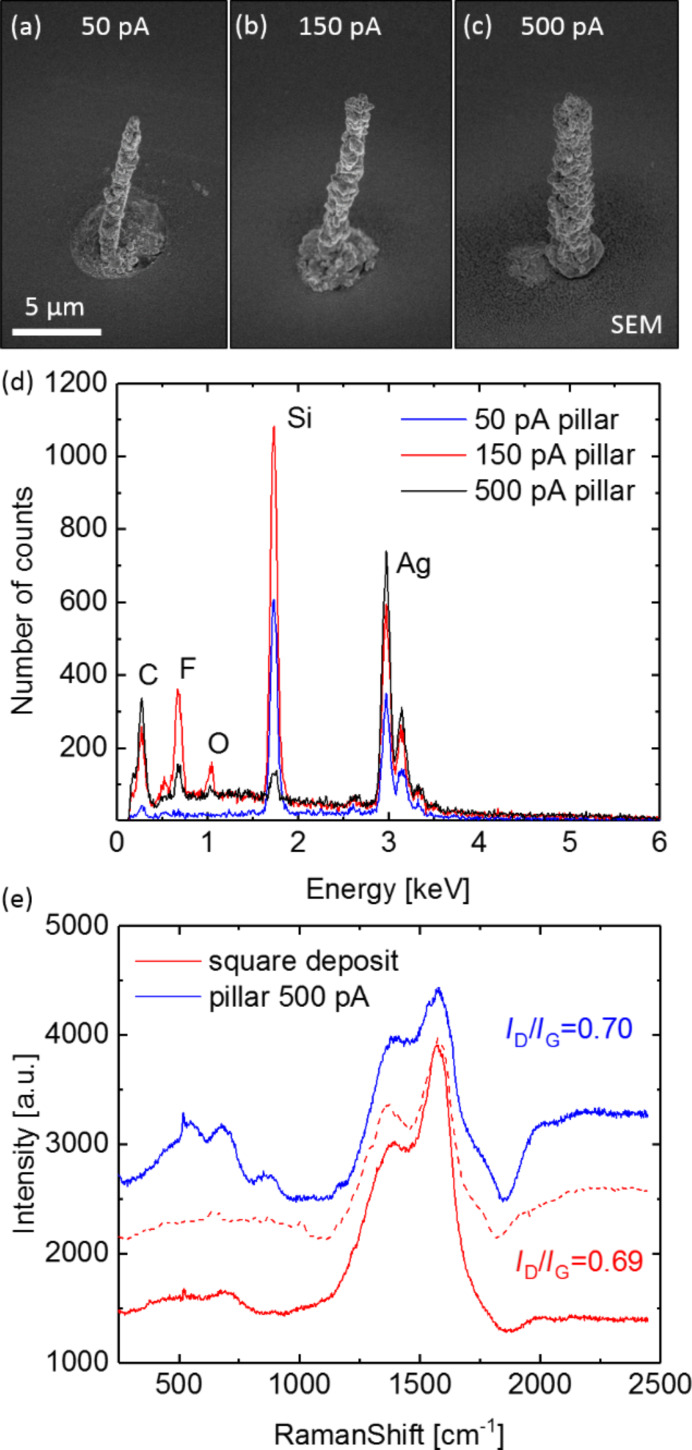
Scanning electron micrographs of single silver pillars obtained after continuous spot irradiation for (a) 50 pA, (b) 150 pA, and (c) 500 pA beam current using AgO_2_F_5_Prop as precursor compound. (d) EDX spectra of all three pillars, (e) Raman spectra of the 500 pA pillar (blue line) and the planar deposit from Figure 1a (red line). For comparison the spectrum of the planar deposit using AgO_2_Me_2_Bu taken from [15] is added (red dotted line).

The SEM images in Figure 3a–c show the results of continuous spot irradiations using AgO_2_F_5_Prop and different beam currents over several hours. Truly three-dimensional nanostructures with high aspect ratios could be achieved. They exhibit different diameters from around 1 µm for 50 pA to 1.5 µm for 150 pA and almost 2.5 µm for 500 pA. The pillar widths correspond to the full width FW (99.9%) of the SE density [9,27] but exceed the typical nanostructure widths of 3D FEBID [1,3]. Interestingly, the halo diameter tends to increase for decreasing beam current. In view of the results presented in Figure 2, this is most probably caused by forward scattering through the nanostructure.

The EDX spectra taken on the apex of the pillars are displayed in Figure 3d. The observed Si peak implies that the EDX spectrum from the primary electrons (tip apex) is superimposed by X-ray signals generated by forward scattered electrons (reaching the substrate). Neglecting that the forward scattered electrons add to the carbon and oxygen signal, a conservative estimate gives 50 atom % of silver. This lower limit for the silver content for the pillars is contrasted by their polycrystalline nature, their Raman activity in Figure 3e, and the higher silver content measured in the planar deposits. Hence, we conclude that the actual silver content is higher and closer to that of the planar deposits. We attribute the observed high silver contents to the low precursor flux and the high reactivity of the used silver carboxylates. A recent study investigated the dependency of the purity of Co–C nanopillars on the growth pressure in cobalt deposition and provided strong hints that indeed a small precursor flux can trigger higher purity but also broader geometrical features [29].

For applications in plasmonics elemental silver is crucial. Here, surface-enhanced Raman scattering being the most popular plasmonic application serves as benchmark. Indeed, the carbon signal intensity for the Raman spectra in Figure 3e is strongly enhanced due to the plasmonic excitation of the silver particles in both deposits [9,27]. The observed enhancement proves the existence of elemental silver. Oxidized silver as well as an atomic carbon–silver mixture would not be resonant in the visible range. The spectra show both the D and G band features that correspond to sp^3^-hybridized and sp^2^-hybridized carbon atoms [30], respectively, in the residual carbon matrix. The matrix is formed by the carbon-based fragments of the precursor molecules. The intensity ratios between the D and G bands (*I*_D_/*I*_G_) were evaluated using a Lorentzian peak fitting, which suggests the formation of nanocrystalline graphite clusters within a disordered carbon matrix in both deposits [30]. The Raman measurements display a very similar carbon configuration for the 500 pA pillar (blue line) and the planar square deposit from Figure 1a (red line). In case of the pillar, the background shows more distinct molecular vibrations, most probably arising from residual precursor fragments. The *I*_D_/*I*_G_ ratios of both deposits are roughly equal to the deposits using AgO_2_Me_2_Bu (red dotted line in Figure 3e).

## Conclusion

Electron beam induced deposition using the two silver carboxylate compounds AgO_2_Me_2_Bu and AgO_2_F_5_Prop was investigated. Both compounds lead to silver deposition with a pronounced halo and a high silver content. Even though the molecular structure of the compounds is very similar, by varying dwell times and currents distinct differences in the deposition became obvious. AgO_2_Me_2_Bu showed strong depletion in the beam center for currents around 500 pA and no correlation of the deposit heights with the dwell time. In contrast, AgO_2_F_5_Prop showed a strongly increased deposition rate in the focal spot of the electron beam for all used currents. Hence, only from this compound 3D silver pillars could be fabricated. The silver pillars show aspect ratios of more than 10:1 for diameters of 1 to 2.5 µm. Furthermore, the carboxylate precursor AgO_2_F_5_ provides for extremely high silver contents of around 74 atom % in the case of planar deposits and for more than 50 atom % of silver (conservative estimate) in case of pillars. It remains to be further investigated how the observed deposition regimes can be used for the tuning of the metal content. The potential of the obtained silver FEBID structures for plasmonics was demonstrated by surface-enhanced Raman scattering. Therefore this study paves the way for plasmonic applications based on direct electron beam writing of silver. Future experiments will comprise the integration of the gas-injection system into a field-emitter electron microscope to achieve sub-100 nm resolution as typically needed for plasmonic structures [17].
